# Intersubjectivity: Conceptual Considerations in Meaning-Making With a Clinical Illustration

**DOI:** 10.3389/fpsyg.2021.715873

**Published:** 2022-01-10

**Authors:** Alexandra Harrison, Ed Tronick

**Affiliations:** ^1^Harvard Medical School, Harvard University, Cambridge, MA, United States; ^2^Cambridge Health Alliance, Cambridge, MA, United States; ^3^Developmental Brain Sciences Program, University of Massachusetts Boston, Boston, MA, United States

**Keywords:** intersubjectivity, human development, face-to-face still-face, autism spectrum disorder, dyadic states of consciousness

## Abstract

This manuscript explores intersubjectivity through a conceptual construct for meaning-making that emphasizes three major interrelated elements–meaning making in interaction, making meaning with the body as well as the mind, and meaning making within an open dynamic system. These three elements are present in the literature on intersubjectivity with a wide range of terms used to describe various theoretical formulations. One objective of this manuscript is to illustrate how such a construct can be useful to understand the meaning-making observed in psychoanalysis, such as in the treatment of a young child on the autistic spectrum. The challenges in establishing an intersubjective state with a child on the autistic spectrum serve to highlight important features of intersubjectivity. As an important background to this clinical illustration, we illustrate the construct with the scientific paradigm of the well-known face-to-face still-face.

## Introduction

We provide a conceptual construct of meaning making that emphasizes three interrelated elements—interactions with others, interactions through bodies as well as minds, and interactions in an open dynamic system–all of which have an established history in the literature on intersubjectivity. The concept of intersubjectivity inherently embraces the importance of interactions among individuals in the process of making meaning, just as there is a line of thinking that emphasizes humans making meaning with their bodies as well as through language. The formal characterization of meaning making as a dynamic systems process is of more recent vintage, although this important perspective also has a history in the literature. The usefulness of this multifaceted conceptual background on intersubjectivity can be illustrated by considering the experimental setting of the Still Face and then by considering a more complex clinical therapeutic setting.

As noted, our conceptualization is framed within the general principles of non-linear systems theory. In our use of intersubjectivity, the exchange of meanings between two individuals is highlighted as potentially co-creating new meanings that are more complex and resource-enhancing than the meanings each individual had previously contributed to the exchange. This model fulfills Prigogene’s first principle of open dynamic systems: Systems must gain resources – in our view meaning–to maintain and expand their organization. Failing that, they dissipate (Prigogine and Stengers, 1984).

This formulation also emphasizes that meaning making is most effective when occurring in interactions between two humans, in intersubjective experiences. That is because the meanings that are exchanged, and the potential co-created new meanings, are more complex than those made by an individual alone. Moreover, intersubjectivity is not the end state of the process. Rather, achieving intersubjective states generates a connection to the other, a trust in oneself and in the other, and a more coherent sense of self in relation to the world (Tronick and Gold, 2020).

This enhanced co-created meaning involves neurosomatic elements, by which we mean bodily elements that are often out of awareness, quite apart from the verbal elements. The multiple sources of meaning-making—of the conscious mind, the dynamic unconscious, the motor system, the endocrine system, the tactile sensory system, and others—create polymorphic forms of meaning that evolve over time and fit only messily together. One of the mysteries of the process is that from this constantly and messily evolving temporal flow of meanings, each individual assembles meanings that allow her to maintain a sense of continuity as a unique individual, a coherent sense of herself in the world (Sander, 2008).

## Meaning Making in Interaction

An important body of research emphasizing interactions among two individuals in meaning making has introduced various terms including Joint Shared Attention, Theory of Mind, and Interaction Theory. It is beyond the scope of this manuscript to consider all this large literature, but it is useful to provide some background on the various studies and nomenclature as a means of acknowledging this critical component of meaning making.

In the 1970’s researchers described young infants’ capacity to share the focus of attention with an adult when prompted by pointing or eye gazing (Scaife and Bruner, 1975). These observations stimulated questions about how infants come to be aware that other minds know theirs and that they can know another’s mind. Bruner posed the question succinctly: “Is it so farfetched that humans know in some crude way from the start that their conspecifics have in common certain experiences of “inner states” like intending or desiring and that in time with the development of sufficient processing capacity they grow more “expert” in reading these experiences and states?” (Bruner, 1995, p. 3). Bruner clarifies his idea of joint visual attention as a “scaffold” for the later emergence of theory of mind. Bruner notes Tomasello’s focus on the importance of the infant’s recognition of intentionality as a critical feature of the scaffold (Tomasello, 1995). Even 1-year old children can distinguish between events that were physically caused, such as dropping something accidentally, or intentional (Poulin-Dubois and Schultz, 1988). Bruner elaborates his view of scaffolding in a 1978 manuscript describing the “active negotiation” in the dialog of a mother and child reading a picture book (Ninio and Bruner, 1978).

The concept of theory of mind was further developed in the false belief studies. Wimmer and Perner (1983) and Baron-Cohen et al. (1985) studied young children’s ability to distinguish between beliefs based on reality and beliefs held in another person’s mind in the Sally Anne or false belief test. In his studies of autism, Baron-Cohen (1995) described “mind blindness” as the inability of autistic individuals to imagine another’s mental state. However, further study of the false belief test revealed that infants and toddlers do in fact demonstrate theory of mind skills when they are given tests appropriate to their developmental level. For example, Onishi and Baillargeon (2005) found that 15-month-old infants looked longer–indicating surprise—at a false belief situation than at a true belief situation involving a hidden toy, and Surian et al. (2007) obtained the same result in an experiment using an animated film about an animal searching for an object.

Tomasello et al. (2007) described how infants will use pointing to influence others’ mental states. In support of Bruner’s idea of scaffolding through social negotiation, O’Madagain and Tomasello (2021, p. 4077) describe a “uniquely human sociolinguistic phenomenon… “joint attention to mental content” through which children develop their rational capacities.

Two theories—theory theory (TT) and simulation theory (ST) both describe indirect processes that use either theoretical inference (TT) or simulation—putting oneself in the other person’s position (ST)—to understand another person’s mental state. Considering these theories, the infant researcher Reddy (2008) describes the impossibility of disembodiment in her description of how babies know minds and proposes a creative elaboration of intersubjectivity in infants.

A significant step forward (and going beyond TT and ST) was developed by Gallagher (2004, 2008) in his Interactive Theory of social cognition. Interactive Theory (sometimes referred to as enactive intersubjectivity) describes ways of achieving an interactive experience through bodily matching and interactive synchrony (Fuchs and De Jaegher, 2009; De Jaegher et al., 2010). This theory is reminiscent of Beebe’s demonstrations of vocal coordination in the mother-infant dyad (Beebe and Lachmann, 2002; Beebe et al., 2005). Gallagher (2013, 2020) further elaborated Interaction Theory in his comprehensive Pattern Theory of Self, in which the self is conceptualized as constituted by multiple characteristics including bodily, experiential, affective, intersubjective and other features (Gallagher and Daly, 2018).

## The Body and Intersubjectivity

We know that humans also make meaning about other human beings not only with their minds but also with their bodies, out of conscious awareness. In his seminal work, Merleau-Ponty (1945) introduced the term intercorporeality to underscore the role of the body in meaning making. Merleau-Ponty elaborated the importance of the bodily experiences in developing pre-conscious understandings of the world and its meaning, a process that is open-ended and always changing (Tanaka, 2015). This process applies to all individuals, including children (Apter et al., 2019). Indeed, in infants and young children–given their lack of language and reflective awareness–Tronick (1980, 2007) has argued that intercorporeality is especially essential in meaning making.

Trevarthen (1974, 2005) was one of the first to describe the intersubjective meaning-making of infants and mothers with their bodies, noting that “even newborn infants… communicate intricately with the expressive forms and rhythms of interest and feeling displayed by other humans… (giving evidence of) purposeful intersubjectivity, or an initial psychosocial state” (Trevarthen and Aitken, 2001, p. 3). Trevarthen refers to the social intelligence of the infant as “a specific human talent—an inherent, intrinsic, psychobiological capacity that integrates perceptual information from many modalities to serve motive states” (ibid, p. 4).

Trevarthen also notes that the infant’s responsiveness to the rhythms of his mother begins before birth—with the infant’s perception of the mother’s heartbeat, and the rhythms and tonalities of her speech and the speech of others in the environment. These perceptual capacities and the infant’s active reciprocal responsiveness prepare him to meet his parents and to know them (Trevarthen and Delafield-Butt, 2013). All this occurs before language (DeCasper and Spence, 1986; Hepper, 1991; Fifer and Moon, 1995; Lacanuet and Schaal, 1996). Trevarthen describes how these capacities support the “emergence and development of active self and other awareness in infancy” (Trevarthen and Aitken, 2001, p. 3). In what he calls his descriptive research, Trevarthen (2015) elaborates the infant’s bodily means of making meaning. He elaborates the infant’s use of the body in intersubjective meaning-making through his work with Malloch, using the term communicative musicality to describe the coordination of time patterns through the body with the purposeful aim of the infant’s movements (Malloch and Trevarthen, 2009).

Porges (2011, 2015, 2020) and Porges et al. (2014) has developed theories about the neural regulation of bodily organs and how they affect behavior and emotional responses in dyadic interaction. Porges (2004) uses the term neuroception to describe the non-conscious system for detecting threats to safety. An example of these neurosomatic processes is the meaning making of the kindling effect on brain neuronal activation that leads to making meaning of a non-threatening event as dangerous (Hofer, 2006; Haglund et al., 2007). Porges’ concept of psychological safety that is communicated by bodily movements is critically important to the clinician.

In children, Snidman et al. (1995) has shown that infants who are shy or inhibited in contrast with uninhibited have different cardiac reactivity patterns to similar events. These cardiac differences are thought to underlie individual differences in behavior and differences in meaning made of the same event. For example, the inhibited children were fearful of a toy robot, whereas the uninhibited children readily played with it. Similar effects on meaning making are found for children in sensory integration clinical work with young children.

Research finds a variety of neurosomatic mechanisms underlying the differences in meaning made of events. Conradt et al. (2015) have shown that at 4 months of age children whose mothers were stressed during pregnancy have poorer attention during face-to-face play and poorer self-regulatory capacities when stressed during the Still-Face paradigm. They found that the behavior and the meaning of the event as more stressful was related to the methylation of the placental gene (NR3C1) that transforms maternal cortisol into cortisone, resulting in greater exposure of the developing fetus to neurotoxic effects of higher levels of cortisol. Thus, fetal experience affected how the infant made meaning of the event after birth.

Regarding intersubjectivity, studies have been done of meaning making with neurosomatic coordination of behavior and gestures (Hofer, 1984; Montirosso et al., 2012, 2014). Interestingly, some of these behaviors were related to sex differences in the infants (Tronick and Cohn, 1989; Weinberg et al., 1998). Tronick interpreted the sex difference as suggesting that girls are better able to modulate their reactivity than boys, producing a more benign meaning for the girls. Intersubjective neurosomatic coordination, or what Ham and Tronick (2009) call relational psychophysiology, is observed in cardiac and parasympathetic activity, and even in brain activation (Feldman et al., 2011; Konvalinka et al., 2011). Many other examples (see, Feldman, 2007; Montirosso et al., 2013, 2015; O’Brien et al., 2013; Tronick and Perry, 2015), substantiate the essential point that the meaning made of an event is related to and affected by underlying neurosomatic systems that are out of awareness–not symbolic or verbal or related in obvious ways to cognitive processes–and that these processes occur between individuals and affect their experience of each other (Van der Kolk, 2009).

## Still-Face Experiment

The Face-to-Face Still-Face paradigm (FFSF; Tronick et al., 1978) is a protocol in which the infant is positioned in an infant seat facing the mother, and the mother is instructed to play with her infant for 2 mins, at which moment she receives a signal to assume an expressionless face. The “Still-Face” condition is maintained for a subsequent 2 mins, after which the mother receives a second signal to resume her original responsive behavior toward her baby. During the still-face episode, infants typically attempt to engage the still-face mother by smiling, gesturing, and vocalizing. When that fails, the infants become distressed and experience physiological arousal–may drool, choke, and spit up. They avert their gaze from the mother and even turn their head away or arch their backs, communicating with their behavior their state of dysregulation and their attempt to manage the relationship through disengagement. In the reunion phase of the protocol, the infants generally regain their positive affect and reengage with their mothers, but often they display at least an initial hesitancy about resuming the social engagement. This experiment has been used to reliably assess infants’ ability to regulate attentional and affective states, as well as qualities of the infant-caregiver relationship (Adamson and Frick, 2003; Mesman et al., 2009; Provenzi et al., 2016). What can the Still Face tell us about intersubjectivity? Infants and caregivers communicate their affect and intention through behavioral exchanges, using facial expression, gesture, and vocalization (Harrison, 2014). With these methods they create coordinated rhythms and other patterns of expression that constitute meanings about their relationship, and from which they also derive meanings about themselves. The mother may derive meanings about the quality of her mothering, about her experiences with her own mother, or other relational meanings about experiences with her infant. She will make meanings with her physiological response to her infant’s behavior and his physical appearance and smell, for example, a stress response to his crying, relaxation when hunger cries stop as he begins to feed. Some of these meanings will be organized by language, such as her assessments of her mothering behavior and her infant’s responses, and these may become conscious memories. Many others will be out of her awareness. The infant is also making meanings with his mind and his body—meanings such as, “I like doing this with my mother,” or “I do not want to do this anymore; I need a break,” or meanings about contented satiety or discomfort in his gut. The infant communicates these meanings to his mother with his behavior. The cluster of meanings in mother and in infant at any particular moment constitutes the state of consciousness (SOC) of each partner (Tronick and Beeghly, 2011). The infant demonstrates various SOC’s in the Still-Face—from the enjoyment of playing in the first 2 mins, to the eagerness to engage followed by distress during the Still-Face, and finally to the pleasure at re-engagement in the reunion phase. But what about the hesitation many infants display at the mother’s initiation of play after the Still-Face?

We think that this hesitation demonstrates the infant’s effort to make meaning of the Still-Face experience. It is as if he is saying to himself, “What just happened? What was that all about? Can I trust this reunion?” We suggest that the Still-Face, among other things, demonstrates the interruption of intersubjectivity held by the infant and mother during the play episode before the signal for mother to become unresponsive. It may be that the mother and infant did not enjoy a relationship with a predominantly positive affective tone before they participated in the still-face experiment, but at least they shared meanings about a repertoire of relational patterns—generating SOC’s—between them (Tronick, 2007). One of these patterns might have been, for example, that when mother is sad, the infant’s smiles may cheer her up, and the infant sees her face relax. In the Still-Face, the infant may cycle through all the behaviors that belong to their way of reconnecting after a disruption, and none are successful (Banella and Tronick, 2019). Without her help, the infant cannot make meaning of the experience she just had with this vitally important person. Since these relational patterns in infant-caregiver relationships are associated with neurosomatic meanings to create complex SOC’s, the infant is disrupted in multiple domains, making the infant’s subjective experience even more powerful.

## Integration Into a Conceptual Formulation of Meaning Making With Application to Psychoanalysis

While studies in cognitive science refer to dynamic systems concepts such as continuous evolving meaning making, our formulation owes a major debt to the important studies of Louis Sander, and we root our theory in infant observation and clinical psychoanalysis (Harrison and Tronick, 2007; Sander, 2008; Harrison and Beebe, 2018). The clinical perspective underlying our theory emphasizes certain features of the interaction such as those of messiness, multiple meaning making, and agency.

Though clinicians’ narratives of their sessions are markedly linear and coherent, the actual interactions between clinician and patient are messy, and because of the multiple meanings or partial meanings that are exchanged between analyst and patient, along with their timing and mode of expression, we cannot predict which will result in an emergent property of the system. In Sander’s view of interactive meaning-making processes as in continual evolution, “a flow of a sequence, of recurrence of expectancy within the recurring exchanges”, (Sander, 2012, p. 168) new meanings emerge that may or may not be instantiated, with resonant effects on other subsystems of the hierarchically organized larger dynamic system. In this sense, the uncoupling described in cognitive science by Fuchs and De Jaegher (2009, p. 471) is not so much a way of “not melting into each other” as it is a moment for each partner to actively claim agency, and a moment for each to incorporate a newly co-created meaning into the self. It is closer to Winnicott’s paradox “the experience of being alone while someone else is present” (Winnicott, 1965, p. 30).

## Inner World of the Self and Dyadic States of Consciousness

Our conceptual formulation provides a place for the private inner world of the self that is not interpretable from the individual’s bodily actions. This inner world holds the complexity but also the continuity of the self that begins in infancy and endures through the lifetime of an individual. Sander presents the paradox between “the uniqueness of each newborn… and each individual’s own particular pathway of development, and the minutiae of events within the flow of interaction between infant and caregiver” and we would add—between the individual and the environment (Sander, 2008, p. 167). The resolution of the paradox, Sander says, is in seeing the developmental process as an integration of “being together with” and “being distinct from” (Sander, 2008, p. 173). In this meaning making process, the emergent properties of the dynamic system of the individual within the larger system of the individual interacting with the world are selected to include new complexity but crucially to maintain the necessary coherence to ensure the continuity of the self.

Tronick’s concept of dyadic state of consciousness (DSC) resembles an intersubjective state, although it is more inclusive than what is typically thought of as intersubjective experience (Tronick et al., 1998). A DSC may occur between individuals when they use behavior to exchange intentions, affects, states of mind, and cognitive meanings with each other. This interaction has the potential to co-create new meanings that in turn can then be appropriated by each individual into their own SOC, their private sense of self, their own inner worlds. Individuals overcome the limitation of self-organizing meaning-making by engaging in dyadic meaning-making, in that way creating intersubjectivity. When an individual’s SOC gains complexity and coherence, the individual undergoes an amplification of self-experience, a sense of emotional and cognitive expansion. Typically, this is pleasurable, but not always.

One might consider that psychoanalysis supports developmental growth through an evolving process of creating a DSC between patient and analyst, which then gets disrupted before continuing on to the creation of the next DSC (Harrison and Tronick, 2011; Tronick and Beeghly, 2011; Harrison and Beebe, 2018; Heller et al., 2019). The potential for creating DSC’s through intersubjectivity is greater in psychoanalysis than in a typical relationship because of the explicit knowledge and experience of the psychoanalyst, the implicit relational knowing of both psychoanalyst and patient, the motivation and capacities of the patient, and the frequency of the sessions—offering many opportunities to make meaning together. However, in contrast with most psychoanalytic thinking, our view of intersubjectivity includes meaning-making with bodily and out of awareness neurosomatic meanings. As we have noted, neurosomatic meanings are made by polymorphic systems operating at multiple levels in the individual. These polymorphic systems of meaning-making include bodily movements to set points of physiological systems, and even genetics and epigenetics, as well as verbal and symbolic communications.

## Psychoanalysis and Intersubjectivity

Psychoanalysis has embraced the concept of intersubjectivity as a way of explaining how one person gains access to another person’s inner world. Led by the Relational School of Psychoanalysis, psychoanalysts have increasingly appreciated the importance of the concept of intersubjectivity, shifting the focus of attention from the inner world of the individual to include the relational matrix (Stolorow et al., 1994; Dunn, 1995; Seligman, 2018). Many see the intersubjective perspective—the idea that the focus of psychoanalysis is the interplay between two subjectivities—as having moved into the foreground of psychoanalytic theory (Stern, 2005; Benjamin, 2013). The literature on intersubjectivity in psychoanalysis is substantial and beyond the scope of this manuscript. We will instead focus on the polymorphic, or polysemic—capable of having multiple meanings–features of intersubjectivity as we use it in psychoanalysis, which extends the intersubjective perspective to include the body and mind of the individual in interaction with the body and mind of the other without disregarding the interaction of the individual with his or her own self, and while always preserving the integrity of the meanings made at each level.

Although psychoanalysis has increasingly introduced ideas about non-verbal communication into psychoanalytic theory, the means of achieving intersubjectivity remains primarily through the exchange of verbal meanings. With few exceptions (Beebe and Lachmann, 2002; Knoblauch, 2005; Harrison, 2014; Harrison and Beebe, 2018; Seligman, 2018), analysts typically describe intersubjective experiences as the verbal trading of evocative images and metaphors in combination with astute self-reflection—again, in thoughts organized by language.

We see the features of dynamic systems theory are particularly helpful to the practicing psychoanalyst in her work. She must contain the complexity of multiple evolving meanings that occur during the course of a clinical session. She must tolerate the uncertainty and variability of her patient’s communications and similarly of her reactions to them. If she settles too quickly on a meaning, she may foreclose alternative meanings that are often represented at the same moment or in the flow of ongoing moments in a complex gesture. For example, an autistic child who expressed positive interest in the analyst but who also perceived her presence as a threat, moved to pick up a toy near where she was sitting, while also averting his gaze and turning his head and torso away. As she waited for the child to initiate another behavioral cue, accepting her not knowing position, the analyst communicated to the child her willingness to give him a turn, to support his agency.

## Influence of Autism on the Process of Meaning Making

Individuals on the autistic spectrum disorder (ASD) have difficulty processing social stimuli, making presuppositions about how other people think and feel, and therefore, creating intersubjective meanings. Baron-Cohen’s false belief studies were motivated by an interest in learning about autism. Baron-Cohen et al. (2001) have more recently shown that factors other than those attributable to ToM may be involved in these difficulties. For example, a significant relevant finding is that ASD individuals have difficulty recognizing vocal cues (Chevallier et al., 2011; Porges et al., 2014). Another important finding is ASD individuals’ diminished social interest (Dawson et al., 1998). It seems logical that if autistic individuals neglect social cues in infancy, their progressive neurodevelopment takes on a different form of organization–one we do not understand, but one that does not support typical social and intersubjective capacity. Social perceptual skills developing during childhood and linked with social cognition, scaffold social skill—or ToM skill–development (Schultz, 2005).

Another theory from cognitive science relating interpersonal problems in autism to intersubjectivity is that the development of Trevarthen’s primary intersubjectivity in ASD individuals is compromised by a basic impairment in the sensory-motor capabilities that young children use to make sense of their connection with others before language (Trevarthen, 2005; Trevarthen and Delafield-Butt, 2013). This basic impairment in bodily interaction with the environment also leads to a central problem of integrating perceptual experience, or a lack of central coherence (Gallagher, 2004). An example of this lack of capacity to integrate perception with context is when a gifted autistic child who learned to read at 2-years old, looked at a picture of the cardinal directions on a new toy with arrows and N, S, E, and W arranged around a circle and said, “That says ‘news’.”

## Background on the Clinical Illustration

Clinical insights into intersubjective processes can be gained through the treatment of the highly heterogeneous group of ASD individuals because of their atypical processing of sensory perceptions, bodily experience. In a clinical vignette of an ASD child, we will illustrate how intersubjective meaning was made between the child and the analyst through implicit awareness of bodily movements, rhythmic coordination, and (what we assume to be) perceptions of internal organs that we include within “neurosomatic” meanings–in concurrence with verbal meaning. Following Trevarthen (2005, 2009, 2015), we emphasize the centrality of the body in our concept of meaning making.

Though we do not have data on all the neurosomatic processes making meaning in this clinical case, the literature documents atypical neurosomatic reactivity of ASD individuals, for example, oxytocin (functioning as anxiolytic) release (Hammock et al., 2012), response to human facial expressions (Nelson, 2012), and the sounds of the human voice (Porges et al., 2014; Porges, 2019). From this evidence we can assume that the child described below makes neurosomatic meaning of his encounter with the analyst in atypical ways. The correspondingly atypical and creative behavioral communications described in the example demonstrate the multiplicity of meanings—the polysemic meaning making–taking part in intersubjective process more strikingly than if language and symbols alone were considered. In this examples, bodily movements used both explicitly and implicitly to make and convey meaning in the relationship illustrate how meaning making evolves in the analytic session. It is not possible, but if it were, we would also explore the neurosomatic processes involved, such as the autonomic and neurohormonal systems.

Our understanding of the behavioral communications in videotape is enhanced by the NCAST scales of infant behavioral cues. These cues, categorized into engagement cues and disengagement cues, have been validated by extensive observations of infant-parent dyads and used in many studies (Farel et al., 1991; Kelly and Barnard, 2000; White-Trout et al., 2013; see also P. Ogden on adult engagement cues, Ogden and Fisher, 2015). The engagement and disengagement cues, while serving as the primary communicators of affect and intention in infancy, persist in later life, although they recede in prominence as language becomes dominant. Behavioral cues are bidirectional, in that each individual in the dyad communicates affect and intention to the other in a back and forth or circular manner. Actually, they could be called double bidirectional in that simultaneously, each individual’s behavioral cues are communicated to his or her own neurosensory system. If the infant communicates engagement to the mother with an open mouth and direct gaze, the mother will typically reciprocate with direct gaze and a smile, her softened facial expression and her gaze not only communicating the desire and intention of engagement to her baby, but also stimulate her own vagal system to generate a flexible sinus rhythm and a feeling of sociability (Porges, 2015). Porges (2015) describes the process of “neuroception” in which the goals and motivations of both inanimate and animate objects are interpreted out of awareness, sending signals to the temporal cortex, creating meanings of threat and safety. In our example, we will elaborate our descriptions by identifying some of the visible NCAST cues.

## Background on the Patient and the Analysis

The clinical example is that of “Hal”, a 3-year-old boy. Dr. A, the analyst, saw him in analytic play sessions four times a week. The session reported here occurred 6 months into the analysis. Dr. A videotaped the sessions and micro analyzed selected sessions, using a modified version of the technique developed by Beebe, in which the second-by-second vocal turns and “action turns” are documented using the Quick Time framework (Harrison and Beebe, 2018). The second-by-second description is reminiscent of Trevarthen’s descriptions of an infant conducting a lullaby or of an infant shaping his mouth and tongue blowing bubbles (Trevarthen, 2005).

Hal had precocious language development, but his speech had the unmelodious quality of ASD individuals. He was interested in his peers but lacked the capacity for reciprocal play and had no real friends in his preschool class. When he had a plan in mind and was unable to accomplish it, he could become agitated and inconsolable. Hal was a gifted child and had an intense interest in–and competence in—reading maps. Dr. A understood this interest as a way Hal made sense of his world in two dimensions.

## Interactions in the Beginning of the Clinical Session

In the session, Hal had the idea of creating maps out of “H-links”, a construction toy. Dr. A had no idea how they would do this, but she joined in his plan with a feeling of pleasant anticipation. The following is what transpires at the beginning of the clinical session.

The segment begins with Dr. A placing the bin of “H-links” on the floor of the playroom. Hal then begins to organize the pieces. Dr. A walks around to a clear space next to Hal and in the first 6 s of the film, settles to the floor. At that moment Hal is gazing down at the H-links and has his back to her. Both his averted gaze and his bodily turn away are NCAST disengagement cues that communicate to Dr. A and to Hal himself his lack of readiness for engagement. Hal does not acknowledge Dr. A’s presence verbally or with his gaze.

At this point, Dr. A moves her hand to her chin. This gesture is a disengagement cue, communicating to Hal and to herself that she should hold back. At second 8, she removes her hand from her chin, looks down, and begins slowly to assemble a small pile of H-links near her. This activity—her activity with the toy but averted gaze–represents a conscious effort on Dr. A’s part to connect with Hal at a distance, in other words, without direct engagement. At second 10, Hal appears to notice an H-link near Dr. A, and moves toward her, communicating an intention to engage (reach in the direction of Dr. A) but again, at a distance (averted gaze). At second 11, Dr. A extends her right leg as if to balance herself. At second 15, Hal brings back the H-link to where he was originally working, and as he leans to the right, Dr. A also moves to the right, continuing her slow, repetitive activity.

At this moment, Dr. A and Hal assume parallel body positions, leaning in the same direction with the same arch of their bodies, and with gaze down. Less than 1 s later, Hal speaks for the first time. He says, “These H-links don’t go together that much.”

## Illustration of Co-Created Meaning Making in This Clinical Example

As Dr. A initially sits down next to Hal, it appears that Hal is not consciously attending to Dr. A’s body movements, and he does not signal an acknowledgment of her approach. The message from Hal is ‘My attention is focused on this toy, and information from the social world is not in my conscious awareness.” Dr. A notices Hal’s lack of acknowledgment and settles into a comfortable position. Consciously, she wants to respect Hal’s agenda and prepares herself to wait until Hal initiates an interaction. However, she also makes an unconscious gesture, touching her hand to her chin, in an NCAST behavioral cue of disengagement. This unconscious gesture appears to indicate Dr. A’s assessment of Hal’s lack of preparedness for engagement and is a communication both to Hal and to herself, indicating, “I will not intrude.” While the meaning of the communication relates to both of them, Dr. A is aware that Hal is not looking at her. This is a moment in an intersubjective process in which Dr. A is attempting to hold Hal in her mind but is also responding to him with her body. Some of the multiple domains of meaning making here are Hal’s bodily activity indicating unreadiness, Dr. A’s bodily sensory processing of Hal’s movements and her own, and Dr. A’s conscious mental activity.

In the next few seconds, Dr. A consciously chooses an alternative way of being with Hal rather than direct engagement. She generates with her own body a rhythm that approximates his small repetitive movements, in that way creating for herself a subjective sense of connection with him. Again, although she understands that he is not looking at her, she has the sense of communicating to him her readiness to engage but also her patience. When Hal moves toward Dr. A, he again appears not to notice her body positioned so closely to him in space, and Dr. A moves away slightly to accommodate his proximity, throwing herself slightly off balance and causing her to extend her leg to stabilize her position. Dr. A’s gesture adds more complexity of meaning. Dr. A has the intention to join with Hal, but she inhibits her initiative because of her awareness of his fragility—how easily he can be disturbed by unanticipated physical closeness. As she becomes aware of his fragility, she creates—with her own bodily instability—a fragility within herself. This is also perhaps an intersubjective moment.

Consciously, Dr. A has the impression that Hal does not have her in mind at all at this point, while in his approach his body seems to be moving toward a connection. Two seconds later, Hal moves away again, and Dr. A and Hal assume symmetrical positions. It is as if their bodies are moving in relation to each other without making conscious or even dynamically unconscious meaning until they randomly achieve a “match” in symmetrical bodily positions at a distance. It is significant that the position is a “match” in the shape of their bodies but not in their gaze. This back and forth movement outside of conscious awareness resembles descriptions of interactions of component parts of a dynamic non-linear system, and one might call their sudden “match” in body positions an emergent property of their dyadic system. The emergent property is a way of being together at a distance and without conscious acknowledgment.

## Commentary on the Clinical Example

This example illustrates the usefulness of the construct of meaning making as a non-linear “messy” process involving bodily as well as verbal interactions between a patient and a therapist, a construct that is not an element of standard psychoanalytic theory (Harrison, 2003, 2014; Harrison and Beebe, 2018). In this context, Dr. A and Hal are communicating a somewhat muted affect—mildly positive but tentative. Dr. A’s intention is to make a connection with Hal, as is Hal’s in a general sense, although it is not clear he has that intention at that moment. Also, although Hal is aware of how challenging it is for him to engage with another person, the meaning he makes of this difficulty is not entirely clear to Dr. A. Most likely, Hal sees the difficulty as originating from outside of himself—“this person is too close and I really don’t like that—something bad is happening to me!”

A more complex formulation of meaning making allows us to put all these meanings, or partial meanings or meanings in evolution, together into a messy dynamic activity between two individuals. The non-linear framework means that we can be comfortable with not knowing what will emerge from this process.

It is likely that Dr. A and Hal will continue to make a connection because they meet together multiple times a week and have a fondness for each other. Yet exactly when and how they will make that connection is not at all clear from the interaction in this example. The non-linear systems theory allows us to accommodate the unpredictability and variability of this moment-to-moment attempt to create intersubjectivity. The “match” Dr. A and Hal make with their bodies assuming symmetrical positions seems comfortable to Hal, and with neither verbal nor visual confirmation, Hal perceives a kind of engagement and initiates verbal communication for the first time.

It is also significant that the verbal communication Hal makes is a symbolic confirmation of the atypical characteristics of their match and the difficulty they have making it: “These H-links don’t go together that much.” Dr. A and Hal now have co-created a shared meaning in words and symbols as well as with their bodies. Though Dr. A thought she was not in Hal’s mind until that moment, something about the interactive process of their bodies moving–perceiving the movement of their own and the other’s bodies moving in space, in relation to each other–allowed them to co-create that shared meaning.

It is also a paradoxical meaning in that they are together with their bodies and yet not with their minds until Hal makes the statement of “these H links don’t go together,” at which point Dr. A takes in the complex meaning of their being together and can acknowledge the truth of what he says. Again, the reference to general principles of dynamic systems theory is helpful in that we can see the intersubjective experience gain in complexity and coherence through the emergent property of the building of connection in multiple domains of meaning making.

## Concluding Remarks

The process of meaning making can be seen as an evolving process of multiple meaning-making activities, simultaneously occurring between two individuals and within each individual. Intersubjectivity, then, can be seen as related to each individual’s state of consciousness, made up of many different meanings one is making of the self, including those in and out of awareness and of the body as well as of the mind, emerging from an active engagement with another’s state of consciousness to create a dyadic state of consciousness.

This interplay could be elaborated to consider the interplay or potential messiness of the meanings between individuals, the polysemic discord of meanings within the individual, and the potential dynamic conflicts engendered in the within and between or the inter- and intra-subjective meanings. Each individual receives the dyadic states of consciousness as a subjective experience of enhancement, much as psychoanalysts describe intersubjective states. The dyadic state of consciousness contributes meaning to each individual’s state of consciousness, but the subjective sense of enhancement does not last, because in the evolving intersubjective process each individual will exercise his or her own agency and the match with the other will be attenuated, until over time another dyadic state of consciousness is created. This process is similar to the moment of meeting described in Sander (2008). In this context, intersubjectivity describes the way humans grow in relationships, from the infant-caregiver relationship to all the other relationships throughout the lifetime.

The clinical example of Dr. A and Hal illustrates meaning making as a dynamic evolving and multifaceted process following a non-linear model that includes interactions between the analyst and the patient. The example also illustrates that the interactions involve bodily as well as verbal processes. The co-creativity is illustrated in the clinical example by Dr. A’s bringing her hand to her chin after having settled her body to the floor next to Hal. The deceptively simple gesture is meaningful in many ways. First, it is a visual cue to Hal that she intends to back off or slow down. In this case, Hal is not looking at her, but it is clear that he is extremely sensitive to the movements of her body, so this meaning may have nonetheless been communicated. Second, with this gesture, Dr. A is out of her awareness reminding herself to back off or slow down. Consciously, she is thinking about being careful not to intrude into the space around him that Hal needs to feel safe; she knows both from her experience with Hal and her experience with other ASD children, that anxiety about social engagement can be triggered by even a slight physical intrusion. This latter meaning may be called Dr. A’s implicit relational knowing about working with ASD children (Boston Change Process Study Group, 2002, 2005; Tronick, 2007). Finally, Dr. A’s physical gesture of touching her face is self-regulating, calming her and relaxing her muscles of facial expression, which will send vagal messages to decrease her heart rate and rate of respiration (Porges, 2015). In the case of Hal, his gaze aversion and the lack of orientation of his body toward Dr. A communicates both to Dr. A and to himself his unreadiness to engage with Dr. A. Yet over time as Dr. A and Hal ‘s second by second gestures continue, they come closer together in the rhythms and positions of their movements. And Hal’s astute comment that “these H-links don’t go together that much” confirms in words their difficulty “going together” and indicates that he has been working hard on preparing himself for a connection, providing the promise of future meaning making between the two of them, albeit one that is dynamic and uncertain.

## Data Availability Statement

The original contributions presented in the study are included in the article/supplementary material, further inquiries can be directed to the corresponding authors.

## Author Contributions

Both authors listed have made a substantial, direct, and intellectual contribution to the work, and approved it for publication.

## Conflict of Interest

The authors declare that the research was conducted in the absence of any commercial or financial relationships that could be construed as a potential conflict of interest.

## Publisher’s Note

All claims expressed in this article are solely those of the authors and do not necessarily represent those of their affiliated organizations, or those of the publisher, the editors and the reviewers. Any product that may be evaluated in this article, or claim that may be made by its manufacturer, is not guaranteed or endorsed by the publisher.
